# The association between prenatal depression and persistent postpartum pharmaceutical opioid use in six US states

**DOI:** 10.1002/pmf2.70105

**Published:** 2025-09-16

**Authors:** Linqing Zheng, Emily S. Miller, Maria W. Steenland

**Affiliations:** ^1^ Brown University School of Public Health Providence Rhode Island USA; ^2^ University of New England College of Osteopathic Medicine Biddeford Maine 04005 USA; ^3^ Department of Obstetrics and Gynecology Division of Maternal Fetal Medicine Warren Alpert Medical School of Brown University and Women & Infants Hospital Providence Rhode Island USA; ^4^ University of Maryland School of Public Health College Park Maryland USA

**Keywords:** persistent postpartum opioid use, PRAMS, prenatal depression

## Abstract

**Introduction:**

Opioids are commonly prescribed to manage postpartum pain. Yet, 1.7%–2.2% of postpartum people develop new persistent use of opioids after receiving a postpartum opioid prescription. Individuals who experience opioid misuse often have mental health comorbidities. A common mental health condition among pregnant individuals is depression. Information about the association between prenatal depression and persistent opioid use postpartum could guide shared decision‐making during counseling, but most available studies rely on insurance claims data, which do not capture non‐prescribed opioids. In this study, we examined the association between prenatal depression and persistent postpartum opioid use in selected US states.

**Methods:**

We used data from the 2019 Pregnancy Risk Assessment Monitoring System (PRAMS) Core Questionnaire, Opioid Supplement, and Opioid Call Back Survey from six states (Kentucky, Louisiana, Massachusetts, Missouri, Pennsylvania, and Utah) to examine the association between prenatal depression and persistent postpartum opioid use (defined as postpartum opioid use for 4 weeks or more) in the United States. The survey captured postpartum use of pharmaceutical opioids, including those prescribed to the respondent and those prescribed to another individual. Unadjusted and adjusted logistic regression models were used to assess the association among postpartum individuals. We calculated marginal effects and presented the study results as percentage point differences with 95% CIs. All models used PRAMS survey weights.

**Results:**

A total of 1680 individuals were included in the study, corresponding to 154,878 birthing people in the United States. Of these individuals, the prevalence of prenatal depression was 18.2%. Among those with prenatal depression, 12.3% (95% CI: 7.3, 20.1) of individuals had persistent postpartum opioid use compared to 3.3% (95% CI: 2.3, 4.7) among individuals without prenatal depression (*p* < 0.001). After adjusting for age group, race/ethnicity, educational level, cesarean delivery, and preterm birth, prenatal depression was associated with a 6.6‐percentage‐point (95% CI: 1.9, 11.3) increase in persistent postpartum opioid use.

**Conclusion:**

Individuals with depression during pregnancy were more likely to develop persistent postpartum opioid use. Recognizing the relationship between prenatal depression and persistent postpartum opioid use may help tailor postpartum pain management and counseling strategies.

## 1 INTRODUCTION

Pain is a common experience among postpartum individuals [1]. The American College of Obstetricians and Gynecologists (ACOG) recommends a stepwise multimodal approach to postpartum pain management. Individuals after vaginal delivery may receive opioid prescriptions as a second step if pain control is not achieved with non‐steroidal anti‐inflammatory drugs (NSAIDs) and acetaminophen [1]. After cesarean delivery, postpartum individuals are advised to take NSAIDs, acetaminophen, and limited opioids for pain management [1].

Evidence suggests that postpartum opioid use is associated with an increased risk of subsequent opioid use [2]. Approximately 2% of people who fill an opioid prescription postpartum develop new persistent opioid use [2]. For individuals with a cesarean delivery, postpartum opioid prescription fills with a supply of three days or more are associated with an increased risk of developing serious opioid‐related events such as opioid use disorder or overdose death [3] In addition, the postpartum overdose death ratio has nearly doubled in the past few years [4]. Identifying potential determinants for persistent postpartum opioid use may prevent serious opioid‐related events.

Depression, which is associated with opioid use in the general population, may be an important determinant of opioid misuse in the postpartum population as well [5, 6, 7]. According to ACOG, approximately 1 in 10 pregnant individuals experience depression [5]. In the maternal population, mental health conditions and opioid use often co‐occur, with approximately 60%–75% of opioid‐dependent pregnant individuals are diagnosed with mood disorders [8, 9]. In addition, evidence has shown the presence of biological interactions between depression and opioids through the body's pain‐relieving system, such that depression heightens the risk of opioid misuse [10, 11, 12]. As a result, pregnant individuals with depression may have a higher risk of opioid misuse and developing new persistent opioid use after childbirth than those without depression. Due to the high prevalence of prenatal depression and the common practice of prescribing postpartum opioids, understanding whether prenatal depression is associated with persistent opioid use could guide opioid counseling practices.

Limited studies have examined the association between prenatal depression and persistent postpartum opioid use. One study using electronic medical records and several studies using insurance claims data have found that pregnant individuals with prenatal depression were more likely to develop persistent postpartum opioid use (Table S1 lists the specific definitions used for persistent opioid use in previous literature) [2, 13, 14, 15]. However, studies that use administrative data to examine this association share some important limitations. First, insurance claims miss depression if the person either did not seek care at all or did not receive a billed service related to depression treatment [16]. In addition, administrative data are likely to overestimate opioid use due to the inclusion of unused pills, which are very common for opioid prescriptions [17, 18]. Moreover, administrative data fail to capture pharmaceutical opioid use when the medication was prescribed to another individual, which leaves out approximately 8.9% of pregnant individuals who used opioids from sources other than their healthcare providers [19]. These measurement issues present important challenges for the interpretation of existing research on the relationship between mental health during pregnancy and persistent postpartum opioid use.

In this study, we add to the existing research using multi‐state and population‐based survey data. Our primary objective is to examine the association between prenatal depression and persistent postpartum opioid use among pregnant individuals in the United States. We hypothesize that prenatal depression is positively associated with postpartum opioid use for 4 weeks or more. This information can inform efforts to increase monitoring of opioid use and depression symptoms during postpartum care.

## 2 MATERIALS AND METHODS

### 2.1 Study design

This is a cross‐sectional study that analyzes the Opioid Supplement and Opioid Call Back Survey (OCBS) data from the 2019 Pregnancy Risk Assessment Monitoring System (PRAMS) dataset. The study sample includes all birthing people who resided in the states of Kentucky, Louisiana, Massachusetts, Missouri, Pennsylvania, and Utah and participated in the Opioid Supplement, OCBS, and the Core PRAMS Questionnaire in 2019. Due to the de‐identified nature of the data, this study is considered not human subjects research by the Brown University Institutional Review Board.

### 2.2 Population and sample

The PRAMS is a population‐based, cross‐sectional survey developed by the Centers for Disease Control and Prevention (CDC). It collects information about the pregnancy, birthing, and postpartum experience, and health outcomes of birthing people in the United States. Approximately 50 jurisdictions (most commonly states) participate annually in PRAMS. Each jurisdiction randomly samples 1000–3000 birthing people annually and administers PRAMS by phone or mail between 2 and 6 months after delivery. In addition, subpopulations of interest, such as individuals who have underweight infants, reside in high‐risk geographic locations, as defined by the PRAMS protocol, or identify as racial and ethnic minorities, can be oversampled and different statistical weighting schemes are implemented accordingly [20].

Seven PRAMS jurisdiction sites participated in both Opioid Supplement and OCBS in 2019 [21]. Among those seven jurisdiction sites, West Virginia's response to the Core Questionnaire was not released due to its unmet 50% response rate criteria [22]. The Opioid Supplement and OCBS questionnaire included questions about maternal pain management [23, 24]. The Opioid Supplement was administered at the same time as the Core PRAMS Questionnaire, whereas OCBS was administered 9–10 months after birth [21]. Both supplemental surveys followed the same distribution and weighting process as the general PRAMS survey. The PRAMS average response rate for OCBS states, excluding West Virginia, was 61.1% [22].

### 2.3 Analytic sample

The study dataset included 1807 respondents from the six PRAMS states that released data for the Phase 8 Core Questions, Opioid Supplement, and OCBS [20]. Of those respondents, we excluded respondents without a response to any of the following questions: “During your most recent pregnancy, did you have any of the following health conditions?”; “Since your baby was born, have you taken or used any of the following prescription pain relievers for any reason?”; and “Since your baby was born, how many weeks or months have you used prescription pain relievers?” [24, 25].

**FIGURE 1:**
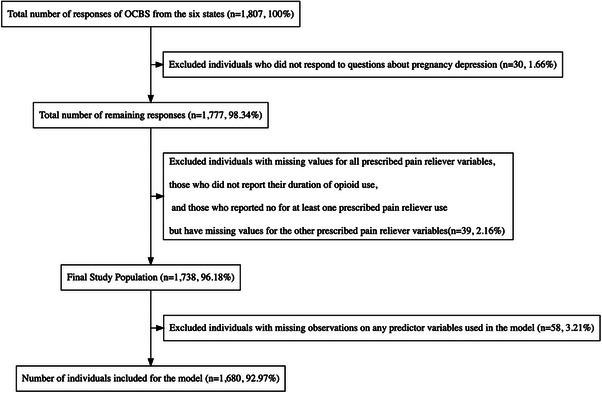
A flowchart that illustrates the selection process of the analytic sample. Number of responses (*n*) and the percentage of responses are reported.

In addition, individuals responding “no” to at least one prescribed pain reliever who were missing responses to any of the other prescribed pain relievers were excluded. We also excluded individuals with missing information on their age, race/ethnicity, educational level, preterm birth, and mode of delivery. Individuals who had missing prenatal insurance status or did not receive prenatal care were included in our model. After the selection procedure shown in Figure 1, we included 1680 participants, or 92.97% of the source population.

### 2.4 Outcome variables

Definitions of persistent postpartum opioid use vary widely in existing literature [2, 14, 15, 26, 27, 28]. See Table S1 for the specific definitions used in previous literature. Since most of the literature used insurance claims data, previous definitions have generally been constructed using the dates and numbers of prescriptions filled. In existing literature, the definition of persistent opioid use ranges from at least two opioid prescription fills within the first 3 months postpartum to at least seven opioid prescription fills within the first 12 months postpartum [28]. The reported median number of opioid tablets included in each prescription fill is 40 5 mg opioid tablets [17], which is equivalent to approximately 10 to 13 days of use [29]. Therefore, the definition of persistent opioid use falls approximately in the range of 20–70 days of opioid use. In this study, we used a definition at the lower end of this standard range, defining persistent opioid use as at least 4 weeks of postpartum opioid use. Persistent postpartum opioid use is meant to capture persons at risk of opioid use disorder. Some individuals in this group have persistent pain and are using opioids as prescribed without negative consequences, while other individuals continue to use opioids to treat non‐surgical pain or for non‐pain symptom management [30]. For our primary outcome, we chose a threshold on the lower end of previously used definitions so that our definition had a greater sensitivity (i.e., captured a larger number of people at risk of opioid misuse). In addition, we conducted a sensitivity analysis where the outcome was defined as postpartum opioid use for 8 weeks or more.

Persistent postpartum opioid use was operationalized using two questions. The first question asked, “Since your baby was born, have you taken or used any of the following prescription pain relievers for any reason?” The respondent could answer “yes” or “no” to the following prescribed pain relievers: hydrocodone, codeine, oxycodone, tramadol, hydromorphone or meperidine, oxymorphone, morphine, and fentanyl [24]. If the individual's response to any of the pain relievers was “yes”, then the duration of postpartum opioid use was assessed by the second question, which asked, “Since your baby was born, how many weeks or months have you used prescription pain relievers?” For the second question, the respondent could report the number of weeks or months they used prescribed pain relievers during their postpartum period. In addition, the respondent had the option to select “less than a week” [24]. We measured postpartum opioid use in weeks and classified “less than a week” as 0.5 weeks of opioid use. If the respondent answered “no” to the first question, the duration of opioid use was coded as zero. We then used the duration of postpartum opioid use information to create one dichotomous variable to represent postpartum opioid use for 4 weeks or more.

### 2.5 Independent variables

Prenatal depression was operationalized by the question “During your most recent pregnancy, did you have any of the following health conditions?” If the individual responded “yes” or “no” to depression, their response was coded as a dichotomous variable [24].

Opioid use during pregnancy was operationalized by the question in the Opioid Supplement “During your most recent pregnancy, did you use any of the following prescription pain relievers?” The respondent could answer “yes” or “no” to the following prescribed pain relievers: hydrocodone, codeine, oxycodone, tramadol, hydromorphone or meperidine, oxymorphone, morphine, and fentanyl [23].

Potential confounding variables were selected from previous literature on the determinants of persistent postpartum opioid use and prenatal depression, including maternal age, race/ethnicity, prenatal insurance types, mode of delivery, educational level, and preterm birth [13, 14, 26, 27, 31].

Maternal age was categorized into four groups (age 19 years or younger, 20–24, 25–34, and 35 years or older). Maternal race and ethnicity were categorized into five groups (non‐Hispanic White, Hispanic, non‐Hispanic Black, non‐Hispanic Asian and Pacific Islander, and non‐Hispanic others). Race and ethnicity were introduced into the model and interpreted as social constructs, reflecting the experience of racism, and not as biological variables. Self‐reported prenatal insurance type was coded as a categorical variable which consisted of Medicaid, private, and none or other insurance. Maternal educational level was categorized into three groups (less than 12 years, 12 years, and greater than 12 years). Mode of delivery and preterm birth were coded as dichotomous variables. The information about the mode of delivery, maternal age, race, ethnicity, preterm birth, and educational level was obtained from the birth certificate file which was included in the PRAMS dataset.

### 2.6 Statistical analysis

We performed all statistical analyses using Stata 18.0 [32]. All analyses used PRAMS survey weights to provide nationally representative estimates. We first report the unweighted frequency and weighted percentage for all demographic characteristics. We also calculated the prevalence of persistent postpartum opioid use by prenatal depression status. In addition, we plotted the duration of opioid use over the postpartum period in the overall population and stratified the prevalence based on the mode of delivery.

This analysis used unadjusted and adjusted logistic regression models and calculated marginal effects. Unadjusted models included each independent variable separately. Adjusted logistic regression models were used to examine the association between prenatal depression and postpartum use for 4 weeks or more, while controlling for potential confounding variables. Results from our analysis are reported in percentage point differences from the reference group (i.e., the difference in the probability of a binary outcome between groups). We present percentage point differences because they are more straightforward to interpret when comparing the differences in the outcomes between groups [33]. For all statistical analyses, we defined the significance level to be 0.05.

### 2.7 Heterogeneity analyses

We conducted an analysis to examine whether there was heterogeneity in the association between prenatal depression and persistent postpartum opioid use by cesarean delivery. Since previous literature has shown that having a cesarean delivery strongly predicts future opioid misuse, we added an interaction term for prenatal depression with cesarean delivery in our adjusted regression models.

### 2.8 Sensitivity analyses

Opioid use during pregnancy may be positively associated with prenatal depression and postpartum opioid use. To better understand whether prenatal depression is associated with persistent postpartum opioid use independent from opioid use during pregnancy, we conducted a sensitivity analysis excluding individuals who used opioids during pregnancy. In secondary analysis, we also examined the association between prenatal depression and postpartum opioid use for 8 weeks or more.

## 3 RESULTS

### 3.1 Sample characteristics

The study sample included 1680 individuals who gave birth in 2019 and participated in the PRAMS survey, the PRAMS Opioid Supplement, and the PRAMS OCBS in the states of Kentucky, Louisiana, Massachusetts, Missouri, Pennsylvania, and Utah. These individuals represented 154,878 birthing people in the six states. In our study sample, most individuals identified as non‐Hispanic white, were 25–34 years old, received a 12‐year education level, had private insurance, underwent vaginal delivery, did not experience preterm birth, and had no depression (Table 1).

**TABLE 1:** Unweighted frequency and weighted percentage of baseline characteristics among study participants.

Baseline characteristics	Unweighted *N*	Weighted % (95% CI)
Age group		
≤19	49	2.8 (1.8, 4.3)
20–24	266	15.7 (13.4, 18.3)
25–34	1021	62.6 (59.1, 66.0)
≥ 35	344	18.8 (16.2, 21.8)
Race and ethnicity		
White, non‐Hispanic	971	69.1 (65.9, 72.2)
Hispanic	231	10.1 (8.3, 12.1)
Black, non‐Hispanic	361	14.9 (12.8, 17.4)
Asian and Pacific Islanders, non‐Hispanic	88	4.6 (3.3, 6.3)
Other, non‐Hispanic	29	1.3 (0.7, 2.4)
Education level (years)		
< 12	156	8.2 (6.4, 10.3)
12	858	50.4 (46.8, 54.0)
> 12	666	41.4 (37.9, 45.1)
Health insurance status		
Medicaid	576	31.3 (28.1, 34.7)
Private	971	59.9 (56.3, 63.4)
None or other	79	4.8 (3.4, 6.6)
Missing or no prenatal care	54	4.0 (2.6, 6.0)
Mode of delivery		
Cesarean	604	34.5 (31.2, 38.0)
Vaginal	1076	65.5 (62.0, 68.8)
Preterm birth		
Yes	236	9.6 (7.9, 11.7)
No	1444	90.4 (88.3, 92.1)
Prenatal depression		
Yes	310	18.2 (15.5, 21.3)
No	1370	81.8 (78.7, 84.5)

**FIGURE 2:**
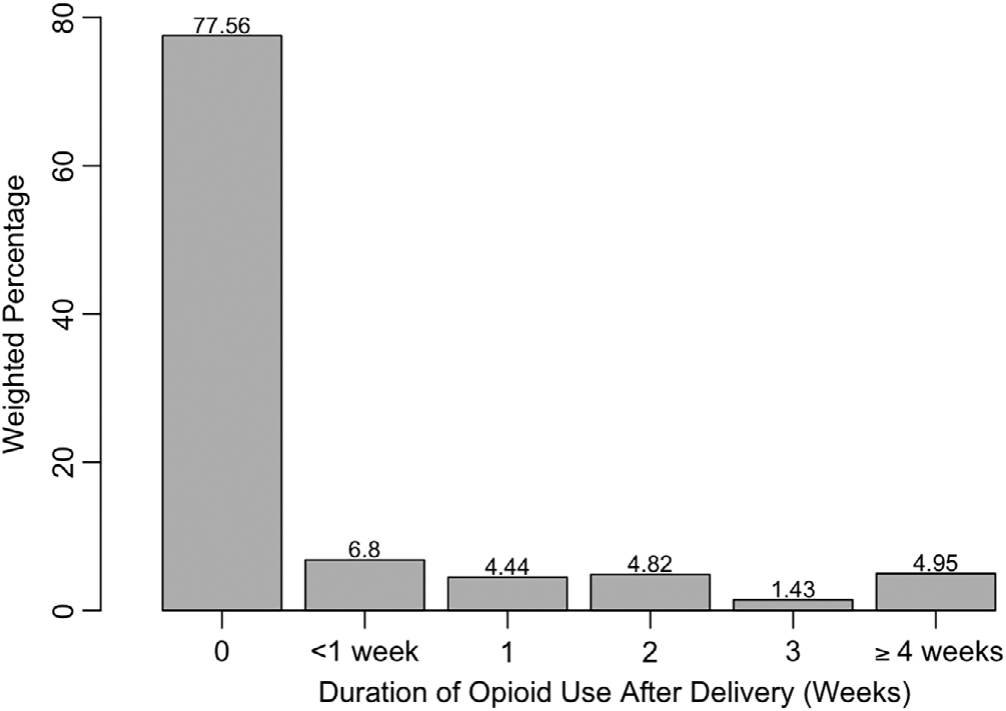
Duration of postpartum opioid use among birthing people in the United States. Data were from the six states that participated in the 2019 PRAMS OCBS. Weighted percentages were calculated using PRAMS survey weights.

Most respondents (77.56%) had not used opioids by the time of the OCBS (9–10 months after childbirth), 6.80% used opioids for less than a week, 4.44% used opioids for 1 week, 4.82% used opioids for 2 weeks, 1.43% used opioids for 3 weeks, and 4.95% used opioids for 4 weeks or more (Figure 2). After stratifying based on the mode of delivery, individuals who underwent cesarean delivery had a higher prevalence of opioid use in all stratified postpartum time periods (less than 1 week, 1 week, 2 weeks, 3 weeks, and 4 weeks or more) compared to individuals who had vaginal birth (Figure S1).

Among individuals with prenatal depression, 12.3% used opioids postpartum for 4 weeks or more. Among individuals without prenatal depression, the prevalence of postpartum opioid use for 4 weeks or more was 3.3% (Table 2). Prenatal depression was associated with a 9.0 percentage point (95% CI: 2.7, 15.4) increase in using opioids postpartum for 4 weeks or more. In the adjusted model, prenatal depression was associated with a 6.6 percentage point (95% CI: 1.9, 11.3) increase in using opioids postpartum for 4 weeks or more, compared to individuals without prenatal depression (Table 3).

**TABLE 2:** Prevalence of persistent postpartum opioid use among persons with and without prenatal depression (*N* = 1680).^a^

	Persistent opioid use ≥ 4 weeks	
	Unweighted *N*	Weighted % (95% CI)
Prenatal depression	26	12.3 (7.3, 20.1)
Without prenatal depression	50	3.3 (2.3, 4.7)

^a^ Pearson's chi‐square test shows a statistically significant difference between the two groups (*p* < 0.001).

**TABLE 3:** Determinants of persistent postpartum opioid use for 4 weeks or more using 2019 PRAMS.^a^

	Persistent opioid use ≥ 4 weeks	
	Unadjusted Percentage Points (95% CI)	Adjusted Percentage Points (95% CI)
	*N* = 1680	*N* = 1680
Prenatal depression		
Yes	9.0 (2.7, 15.4)	6.6 (1.9, 11.3)
No	Ref	Ref
Age group		
≤19	Ref	Ref
20–24	3.4 (−5.9, 12.7)	0.7 (−8.7, 10.2)
25–34	−0.4 (−8.9, 8.1)	−0.4 (−9.4, 8.6)
≥35	−1.8 (−11.0, 7.4)	−2.3 (−11.3, 6.8)
Race and ethnicity		
White, non‐Hispanic	Ref	Ref
Hispanic	−1.7 (−5.8, 2.3)	−1.9 (−5.8, 2.1)
Black, non‐Hispanic	2.5 (−1.9, 6.9)	0.8 (−2.5, 4.2)
Asian and Pacific Islanders, non‐Hispanic	−2.1 (−5.5, 1.2)	−0.7 (−5.2, 3.7)
Other, non‐Hispanic	−0.2 (−6.3, 5.9)	0.5 (−6.2, 7.2)
Education level (years)		
<12	Ref	Ref
12	2.2 (−3.2, 7.7)	0.7 (−5.8, 7.2)
>12	−3.7 (−8.5, 1.2)	−4.1 (−10.4, 2.2)
Health insurance status		
Medicaid	Ref	Ref
Private	−2.0 (−5.7, 1.7)	1.5 (−2.1, 5.0)
None or Other	−2.2 (−10.0, 5.6)	−2.0 (−6.3, 2.4)
Missing or No prenatal Care	−4.1 (−8.4, 0.3)	−2.0 (−5.7, 1.8)
Mode of delivery		
Cesarean	7.8 (3.8, 11.8)	8.2 (4.6, 11.9)
Vaginal	Ref	Ref
Preterm birth		
Yes	2.7 (−2.8, 8.1)	0.2 (−3.5, 3.9)
No	Ref	Ref

^a^ Unadjusted and adjusted multivariable logistic regression models estimated the percentage point difference of each baseline characteristic on persistent postpartum opioid use. PPs and 95% CI were reported. The reference value was shown as Ref for adjusted PPs. All analyses were weighted by PRAMS survey weight.

### 3.2 Heterogeneity analysis

We found no difference in the association between prenatal depression and persistent postpartum opioid use when comparing individuals who had vaginal birth to individuals with cesarean delivery [−0.1 percentage points (95% CI: −5.6, 5.3)] (Table S2).

### 3.3 Sensitivity analyses

After excluding individuals with opioid use during pregnancy, the prevalence of postpartum opioid use was 4.7 percentage points higher (95% CI: −0.1, 9.6) among those with prenatal depression, although this did not reach statistical significance (Table S3). Prenatal depression was also associated with an increase in postpartum opioid use for 8 weeks or more [4.7 percentage points (95% CI: 1.1, 8.4)] (Table S4).

## 4 DISCUSSION

### 4.1 Principal findings

Using data from population‐representative survey data from six states, we found a significant association between prenatal depression and persistent postpartum opioid use in both unadjusted and adjusted models. We did not observe any differences in the association when comparing based on the mode of delivery. After excluding individuals who used opioids during pregnancy, prenatal depression was still associated with an increased point estimate for postpartum opioid use by almost 5 percentage points (though the *p* value was 0.055), indicating that our results were not fully driven by prenatal opioid use. The association was also statistically significant when we examined a longer duration of postpartum opioid use, suggesting our results were robust to alternative definitions of the study outcome.

### 4.2 Results of the study in the context of existing literature

Our findings are consistent with prior literature which has shown that prenatal depression is positively associated with persistent postpartum opioid use [2, 13, 15, 27]. A previous cohort study that used 2017–2019 medical record data showed that antepartum depressive symptoms were a strong predictor of high levels of postpartum opioid use, which was defined as any opioid use after a vaginal delivery and the top quartile of total opioid use after cesarean delivery [13]. Another cohort study that used insurance claims data found that individuals with a depression diagnosis were more likely to have at least 2 fills of opioid prescriptions within the first 3 months postpartum [15]. Other studies have examined broader exposures, such as any prenatal mood disorder or the use of antidepressants during pregnancy, and found a significant association between psychiatric diagnoses with persistent postpartum opioid use among opioid naïve populations who underwent cesarean delivery.

### 4.3 Clinical implications

Several potential mechanisms may explain the positive association between prenatal depression and persistent postpartum opioid use. First, individuals who are depressed often have a higher pain sensitivity, which can lead them to require more prescription pain medications [10, 11, 12]. Second, the brain region that responds to opioids also plays a role in managing mood and stress. This multifunctionality may allow short‐term opioid use to alleviate depressive symptoms [12, 34]. Furthermore, pregnancy may increase individuals’ emotional burdens, and individuals with depression often experience difficulties managing their stress. Since opioids may be used as a coping mechanism for stressful events, individuals with depression may be at a higher risk of opioid misuse [35].

Our study results suggest several potential ways to improve maternal health outcomes. If prenatal depression is a risk factor for persistent postpartum opioid use, identifying and managing depressive symptoms during pregnancy may help individuals limit opioid use for non‐medical purposes. If opioid use is necessary to manage postpartum pain, shared decision‐making and personalized prescribing based on medication needs can be implemented to support individuals with prenatal depression to use opioids safely [36].

In addition, we found that prenatal depression is similarly associated with persistent opioid use, irrespective of mode of delivery. Since opioids are rarely prescribed after a vaginal delivery, this finding highlights the potential that opioids prescribed for postpartum pain management may not be the sole contributors to persistent opioid use. Therefore, assessments of access to other sources of pharmaceutical opioids may be important in preventing persistent postpartum opioid use.

### 4.4 Strengths and limitations

To the best of our knowledge, this study is the first study that investigates the relationship between prenatal depression and persistent postpartum opioid use using population‐representative survey data from a multi‐state sample capturing all pharmaceutical opioids. The relationship between depression and opioid use may be unique in the postpartum population because a large share of the population experiences an acute need for pain relief, is likely to be prescribed opioids, and has high rates of new and ongoing depression [1, 2, 37]. The postpartum period is also a high‐risk period for mental health conditions, including suicide and substance use–related overdose death, which are among the leading underlying causes of maternal mortality [38]. Therefore, understanding the specific relationship between prenatal depression and postpartum opioid use is an important part of improving US maternal health.

Several limitations should be taken into consideration when interpreting our analysis. First, since our study design is observational and no causal inference frameworks are used, causality cannot be assumed for any of the associations in our study. Second, our data comes from self‐reported surveys, and using opioids is often stigmatized; therefore, the data may underestimate opioid use. Third, as pre‐pregnancy opioid use was not assessed by the study's data sources, we could not explore the relationship between opioid use before pregnancy, prenatal depression, and opioid use postpartum. Fourth, because the study's survey data only capture the use of pharmaceutical opioids, we may have misclassified some people who used illicit opioids postpartum. Though effective management of prenatal depression is a potential strategy to mitigate the influence of depression on postpartum opioid use, we have limited data on depression treatment and thus were not able to explore this possibility in this study. Furthermore, our study sample size does not provide sufficient statistical power to detect small or moderate differences in the association between prenatal depression and persistent postpartum opioid use between modes of delivery.

## 5 CONCLUSIONS

We found a positive association between prenatal depression and persistent postpartum opioid use. This finding suggests the potential benefit of identifying and treating prenatal depression in decreasing the risk of persistent postpartum opioid use. Future work is needed to examine whether the significant association between prenatal depression and persistent postpartum opioid use exists when stratified based on the mode of delivery.

## CONFLICT OF INTEREST STATEMENT

The authors declare no conflicts of interest.

## FUNDING INFORMATION

The authors did not receive any funding for this research.

## Supporting information



Supporting Information

## Data Availability

The data that support the findings in this study are available from CDC Pregnancy Risk Assessment Monitoring System (PRAMS). Interested researchers may access the data through the PRAMS Automated Research File (ARF) web portal after giving consent to the PRAMS Data Sharing Agreement.
