# Supplementary material for: The association between prenatal depression and persistent postpartum pharmaceutical opioid use in six US states
Source: Pregnancy (Hoboken). 2025 Sep 16;1(5):e70105. doi: 10.1002/pmf2.70105 (PMC13344678; doi:10.1002/pmf2.70105)
Supplement: Supplementary file 1 [file PMF2-1-e70105-s001.docx]

**Supporting Information & Appendices**

Figure S1. Duration of postpartum opioid use among individuals stratified based on the mode of delivery

Table S1. Definitions of persistent postpartum opioid use from previous literature.

| Previous literature | Definition |
| --- | --- |
| Sudhof et al.^1^ | any opioid use after vaginal birth or the top quartile of total opioid use after cesarean delivery |
| Peahl et al.^2,3^ | at least one opioid prescription for each period between 4 to 90 days and 91 to 365 days after discharge |
| Nidey et al.^4^ | having at least two opioid fills within the first 90 days postpartum |

Table S2. Percentage point difference of postpartum opioid use for four weeks or more after adding the interaction term for cesarean delivery and prenatal depression*

|  | Persistent opioid use ≥ 4 weeks (N=1,680)  Adjusted Percentage Points (95% CI) |
| --- | --- |
| Prenatal depression |  |
| Yes | **6.8 (0.1, 13.4)** |
| No | Ref |
| Age group |  |
| ≤19 | Ref |
| 20-24 | 0.8 (-8.7, 10.2) |
| 25-34 | -0.3 (-9.3, 8.6) |
| ≥35 | -2.3 (-11.2, 6.7) |
| Race and ethnicity |  |
| White, non-Hispanic | Ref |
| Hispanic | -1.9 (-5.8, 2.0) |
| Black, non-Hispanic | 0.8 (-2.6, 4.2) |
| Asian and Pacific Islanders, non-Hispanic | -0.7 (-5.2, 3.7) |
| Other, non-Hispanic | 0.5 (-6.2, 7.3) |
| Education level (years) |  |
| <12 | Ref |
| 12 | 0.7 (-5.9, 7.3) |
| >12 | -4.1 (-10.4, 2.2) |
| Health insurance status |  |
| Medicaid | Ref |
| Private | 1.5 (-2.1, 5.0) |
| None or Other | -2.0 (-6.3, 2.4) |
| Missing or No Prenatal Care | -2.0 (-5.7, 1.7) |
| Mode of delivery |  |
| Cesarean | **8.3 (3.8, 12.8)** |
| Vaginal | Ref |
| Preterm birth |  |
| Yes | 0.2 (-3.5, 3.9) |
| No | Ref |
| Interaction: cesarean delivery x prenatal depression |  |
| Yes | -0.1 (-5.6, 5.3) |
| No | Ref |

*Adjusted multivariable logistic regression models were implemented to estimate the percentage point difference of each baseline characteristic on persistent postpartum opioid use with the addition of an interaction term between prenatal depression and cesarean delivery. Percentage point differences (PP) and 95% CI were reported. The reference value was shown as Ref for adjusted PPs. All models were weighted by PRAMS survey weights. Estimates that had p-values that were less than the defined significance level ($\alpha=0.05$) were bolded.

Table S3. Percentage point difference of persistent postpartum opioid use for four weeks or more among individuals with no opioid use during pregnancy*

|  | Persistent opioid use ≥ 4 weeks (N=1,451)  Adjusted Percentage Points (95% CI) |
| --- | --- |
| Prenatal depression |  |
| Yes | 4.7 (-0.1, 9.6) |
| No | Ref |
| Age group |  |
| ≤19 | Ref |
| 20-24 | 0.5 (-10.5, 11.4) |
| 25-34 | -0.9 (-11.3, 9.6) |
| ≥35 | -2.2 (-12.4, 8.1) |
| Race and ethnicity |  |
| White, non-Hispanic | Ref |
| Hispanic | -1.2 (-5.8, 3.4) |
| Black, non-Hispanic | 1.1 (-2.7, 5.0) |
| Asian and Pacific Islanders, non-Hispanic | -1.8 (-6.0, 2.3) |
| Other, non-Hispanic | -2.0 (-7.4, 3.4) |
| Education level (years) |  |
| <12 | Ref |
| 12 | -1.5 (-10.8, 7.7) |
| >12 | -6.0 (-15.0, 3.0) |
| Health insurance status |  |
| Medicaid | Ref |
| Private | 2.3 (-1.5, 6.0) |
| Missing or No prenatal care | -1.7 (-5.4, 1.9) |
| Mode of delivery |  |
| Cesarean | **7.2 (3.3, 11.0)** |
| Vaginal | Ref |
| Preterm birth |  |
| Yes | -0.1 (-4.2, 4.0) |
| No | Ref |

* Adjusted multivariable logistic regression models were implemented to estimate the percentage point difference of each baseline characteristic on persistent postpartum opioid use among individuals with no opioid use during pregnancy. The “none or other insurance” category variable was removed from the insurance because the new study sample had no individuals in this insurance category. Percentage point differences (PP) and 95% CI were reported. The reference value was shown as Ref for adjusted PPs. All models were weighted by PRAMS survey weights. Estimates that had p-values that were less than the defined significance level ($\alpha=0.05$) were bolded.

Table S4. Percentage point difference of persistent postpartum opioid use for eight weeks or more*

|  | Persistent opioid use ≥ 8 weeks (N=1,626)  Adjusted Percentage Points (95% CI) |
| --- | --- |
| Prenatal depression |  |
| Yes | **4.7 (1.1, 8.4)** |
| No | Ref |
| Age group |  |
| ≤19 | Ref |
| 20-24 | 0.9 (-1.4, 3.1) |
| 25-34 | 1.2 (-0.8, 3.2) |
| ≥35 | 1.5 (-1.6, 4.5) |
| Race and ethnicity |  |
| White, non-Hispanic | Ref |
| Hispanic | -0.3 (-2.0, 1.4) |
| Black, non-Hispanic | 0.7 (-1.5, 2.9) |
| Asian and Pacific Islanders, non-Hispanic | 0.4 (-3.0, 3.8) |
| Other, non-Hispanic | 0.5 (-4.5, 5.4) |
| Education level (years) |  |
| <12 | Ref |
| 12 | 0.9 (-2.0, 3.8) |
| >12 | -1.6 (-3.9, 0.7) |
| Health insurance status |  |
| Medicaid | Ref |
| Private | -0.1 (-2.2, 2.1) |
| None or Other | -1.4 (-3.5, 0.6) |
| Mode of delivery |  |
| Cesarean | **3.6 (1.0, 6.3)** |
| Vaginal | Ref |
| Preterm birth |  |
| Yes | 0.6 (-1.9, 3.1) |
| No | Ref |

* Adjusted multivariable logistic regression models were implemented to estimate the percentage point difference of each baseline characteristic on persistent postpartum opioid use for eight weeks or more. The “missing or no prenatal care” category was removed from the insurance variable because the new study sample had no individuals in this insurance category. Percentage point differences (PP) and 95% CI were reported. The reference value was shown as Ref for adjusted PPs. All models were weighted by PRAMS survey weights. Estimates that had p-values that were less than the defined significance level ($\alpha=0.05$) were bolded.

**Reference**

1.	Sudhof LS, Gompers A, Hacker MR. Antepartum depressive symptoms are associated with significant postpartum opioid use. *Am J Obstet Gynecol MFM*. 2023;5(8):101009. doi:10.1016/j.ajogmf.2023.101009

2.	Peahl AF, Dalton VK, Montgomery JR, Lai YL, Hu HM, Waljee JF. Rates of New Persistent Opioid Use After Vaginal or Cesarean Birth Among US Women. *JAMA Netw Open*. 2019;2(7):e197863. doi:10.1001/jamanetworkopen.2019.7863

3.	Peahl AF, Morgan DM, Dalton VK, et al. New persistent opioid use after acute opioid prescribing in pregnancy: a nationwide analysis. *Am J Obstet Gynecol*. 2020;223(4):566.e1-566.e13. doi:10.1016/j.ajog.2020.03.020

4.	Nidey N, Carnahan R, Carter KD, et al. Association of Mood and Anxiety Disorders and Opioid Prescription Patterns Among Postpartum Women. *Am J Addict*. 2020;29(6):463-470. doi:10.1111/ajad.13028
